# Supplementary material for: Folding and Stabilization of Native-Sequence-Reversed Proteins
Source: Sci Rep. 2016 Apr 26;6:25138. doi: 10.1038/srep25138 (PMC4844985; doi:10.1038/srep25138)
Supplement: Supplementary Information [file srep25138-s1.doc]

# Folding and Stabilization of Native-Sequence-Reversed Proteins

Yuanzhao Zhang^1,2^, Jeffrey K Weber^2^, Ruhong Zhou^*,1,2,3^

*1. Department of Physics, Zhejiang University, Hangzhou 310027, China*

*2. Computational Biological Center, IBM Thomas J. Watson Research Center, Yorktown Heights, NY 10598, USA*

*3. Department of Chemistry, Columbia University, New York, NY 10027, USA*

*Corresponding author, E-mail: [ruhongz@us.ibm.com](mailto:ruhongz@us.ibm.com)


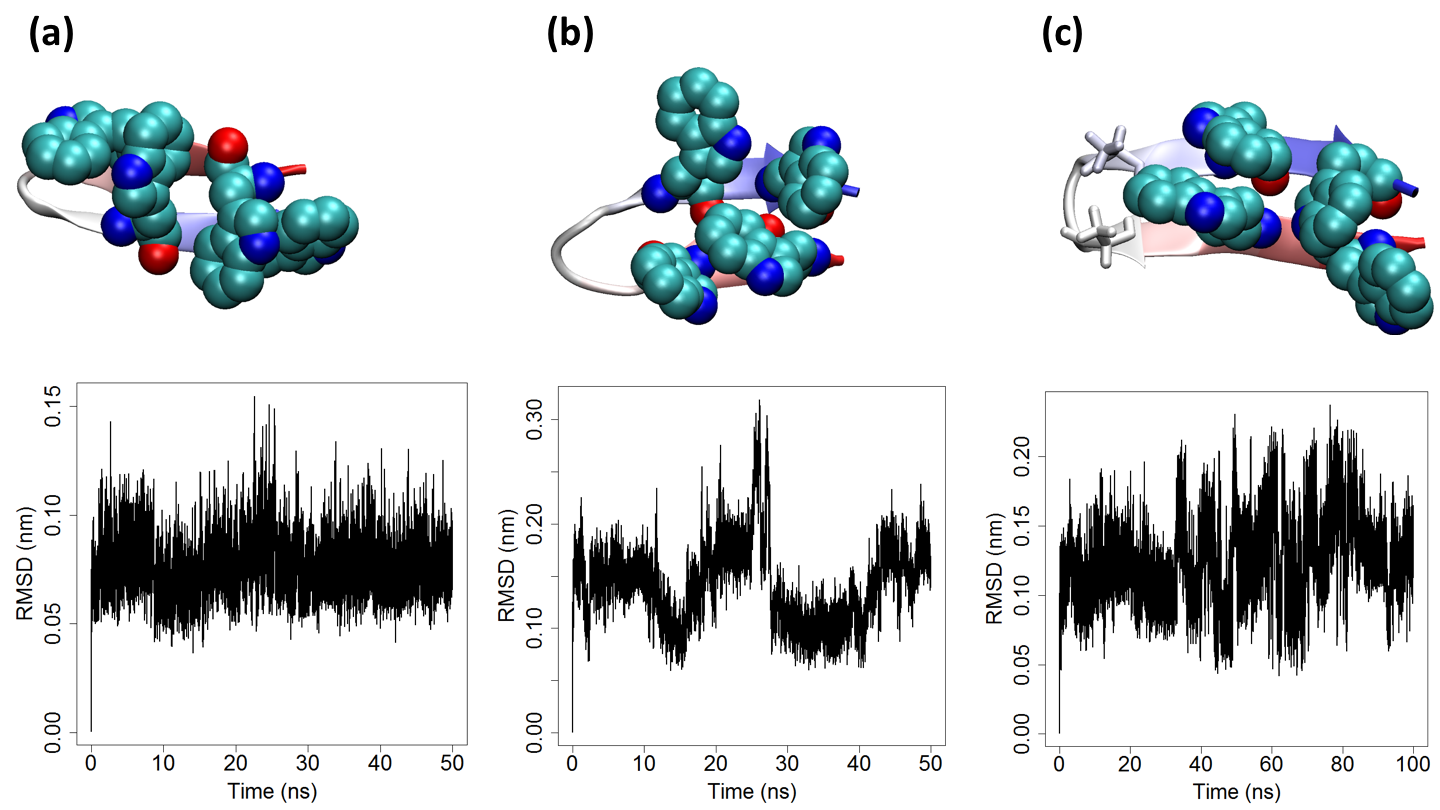


Figure S1: Predicted structures and backbone RMSDs for (a) native Trpzip2 (SWTWENGKWTWK), (b) sequence-reversed Trpzip2 (KWTWKGNEWTWS), and (c) the modified sequence-reversed Trpzip2 (KWTWK**A**GN**A**EWTWS). The plotting scheme mirrors that used in Fig. 1.


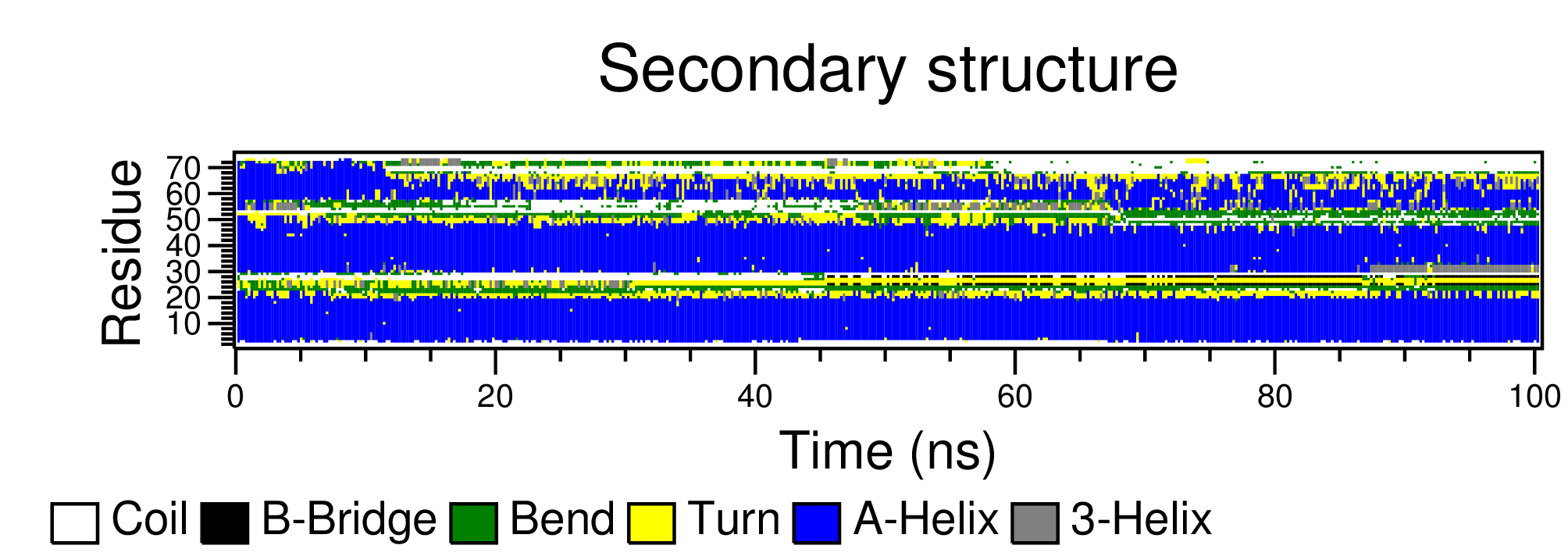


Figure S2: Secondary structural components of sequence-reversed α3D during 100 ns of MD simulation.

| Design | Modification | Sequence | Simulation Summary |
| --- | --- | --- | --- |
| A1 | Insert DE | SPPPRGSSPGGD**DE**KLWQIYLN | Unstable; coil too long |
| A2 | Insert DE  Mutate S to P | **P**PPPRGSSPGGD**DE**KLWQIYLN | Unstable; coil too long |
| B1 | Switch W&L  Mutate S to P | **P**PPPRGSSPGGDK**WL**QIYLN | Relatively stable, but the α-helix is largely destroyed |
| B2 | Switch W&L | SPPPRGSSPGGDK**WL**QIYLN | Relatively stable, but the prolines pack against the tyrosine |
| B2’ | Switch W&L | SPPPRGSSPGGDK**WL**QIYLN | Relatively stable, but the α-helix is largely destroyed |
| B3 | Switch W&L  Mutate L to A | SPPPRGSSPGGDK**WL**QIY**A**N | Relatively stable, but key residues are packed against the tyrosine |
| B4 | Switch W&L  Mutate Y to A | SPPPRGSSPGGDK**WL**QI**A**LN | Unstable; the α-helix melts away |
| C1 | Mutate Y to A | SPPPRGSSPGGDKLWQI**A**LN | Unstable; the α-helix melts away |
| C2 | Mutate W to F | SPPPRGSSPGGDKL**F**QIYLN | Unstable; key residues not well packed |
| C3 | Mutate W to F  Switch F&L | SPPPRGSSPGGDK**FL**QIYLN | Unstable; the α-helix melts away |

Table S1: Ten distinct sequence modification attempts for stabilizing sequence-reversed Trp-cage. The red bold letters indicate the nature of mutations performed within the reverse sequence SPPPRGSSPGGDKLWQIYLN. Some relatively stable sequences emerge from these modifications, but none of the sequences adopt a native-like packing mode.


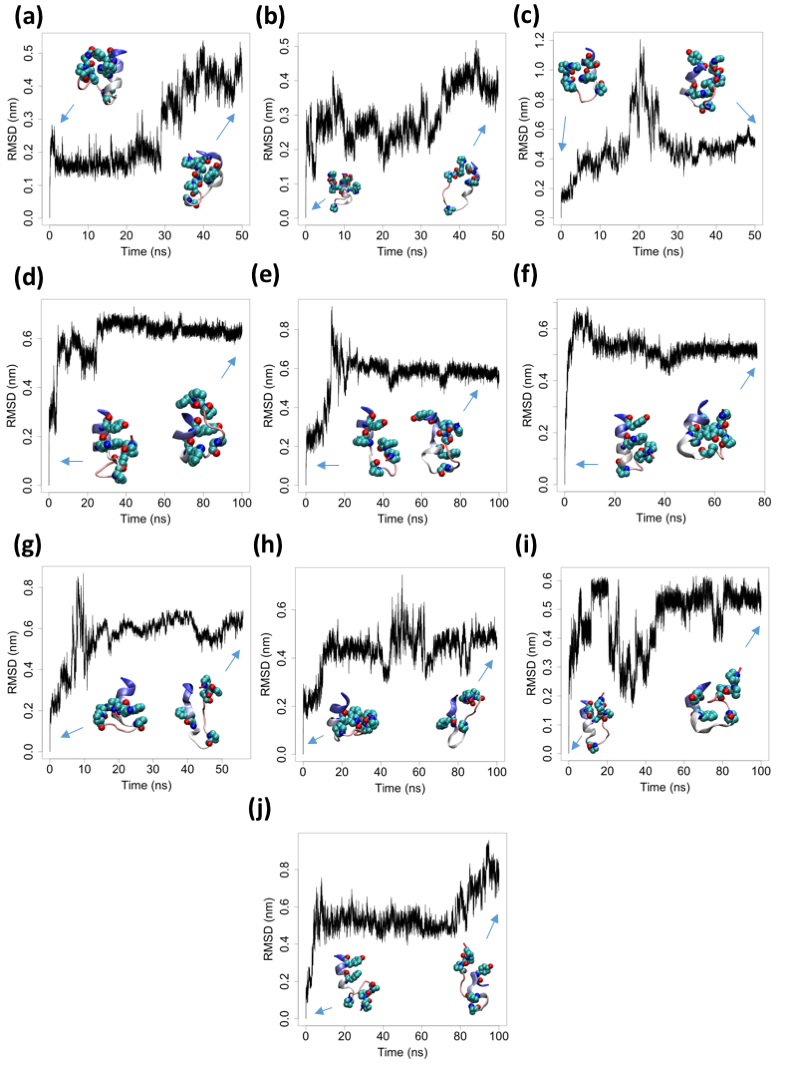


Figure S3: Backbone RMSDs of the modified, sequence-reversed Trp-cages (as tabulated in Table S1) presented alongside snapshots of initial and final protein structures.

Table S1 and Fig. S3 summarize the sequence modifications performed in an attempt to stabilize sequence-reversed Trp-cage. Inspired by our success with β-hairpins, we first implemented analogous strategies (A1 and A2) on Trp-cage by inserting two amino acids at the intersection of the α-helix and
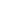
-helix (here, we insert aspartic acid and glutamic acid, as suggested by PyRosetta). These two residues were added in order to endow the α-helix with an additional half-turn, ideally counteracting the geometrical change induced by the inversed chirality and facilitating packing between Trp6 and the sequence’s prolines. However, these modified sequences were unable to give rise to a stable native-like fold, likely because the inserted amino residues introduce a relatively large void into the structure that is detrimental to the packing of the core. We thus moved on to another class of modification (B1 to B4) wherein the positions of Trp6 and Leu7 are switched, hopefully facilitating hydrophobic packing without introducing a void into the structure. This strategy yields many sequences with relatively stable structures, but none recover a native-like fold. As noted in the Trp-cage section of the paper, the directional change of Trp6 caused by the reversal operation poses strict geometrical constraints on possible packing patterns. In particular, the native packing pattern will suffer from severe steric clashes. To alleviate this problem, we tried to mutate away the bulky Leu2 and Tyr3 one turn above Trp6 (B3 and B4) to encourage improved proline packing. Unfortunately, Tyr3 seems to be crucial to the stabilization of the Trp-cage structure, as the α-helix melts away within 10 ns without it. Our final attempt was also consistent with the packing facilitation line of thinking: we mutated Trp6 into the smaller but also highly hydrophobic phenylalanine (C2 and C3). Despite all of these attempts to stabilize the sequence-reversed Trp-cage, the native-like fold proves extremely hard to restore. Taking into account the restrictive geometrical constraints introduced by the reversed chirality, it is clear that the small size of Trp-cage and the precise packing required by the native fold makes it difficult to find a simple sequence modification that can guide the sequence-reversed Trp-cage back into a stable, native-like fold.


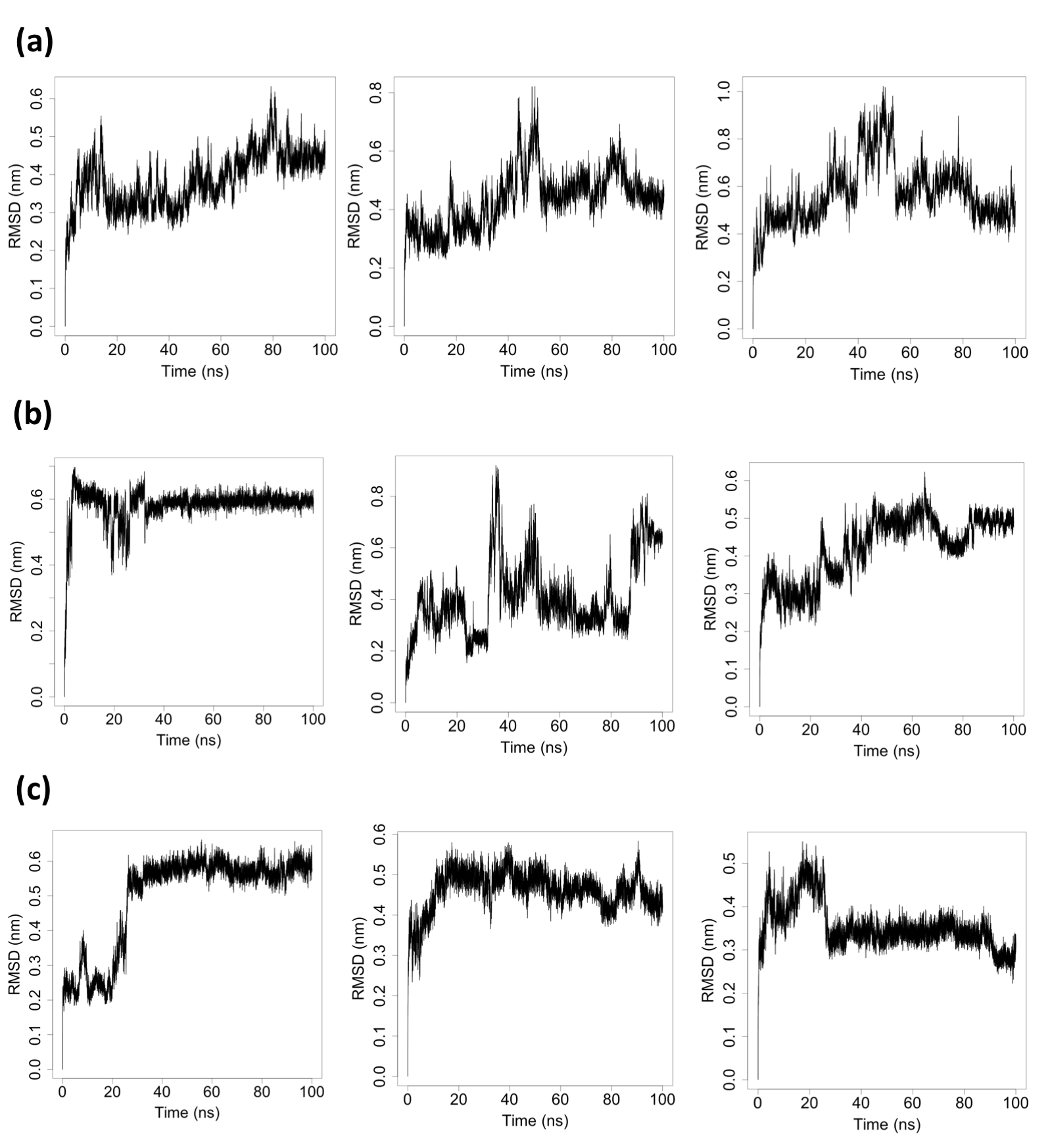


Figure S4: Backbone RMSDs of random realizations of the native sequences of (a) α3D, (b) the C-terminus β-hairpin of protein G, and (c) Protein G in 100 ns MD simulations. The large fluctuations in the time traces demonstrate that those random sequences do not have stable, well-defined structures. Two minor exceptions are the random β-hairpin sequence shown in b1 and the random Protein G sequence presented in c1: both systems enter relatively stable states after 50 ns. More detailed analysis in Fig. S5 reveals that their secondary structures are mostly composed of coils and bends which bear little resemblance to the structures adopted by native sequences.


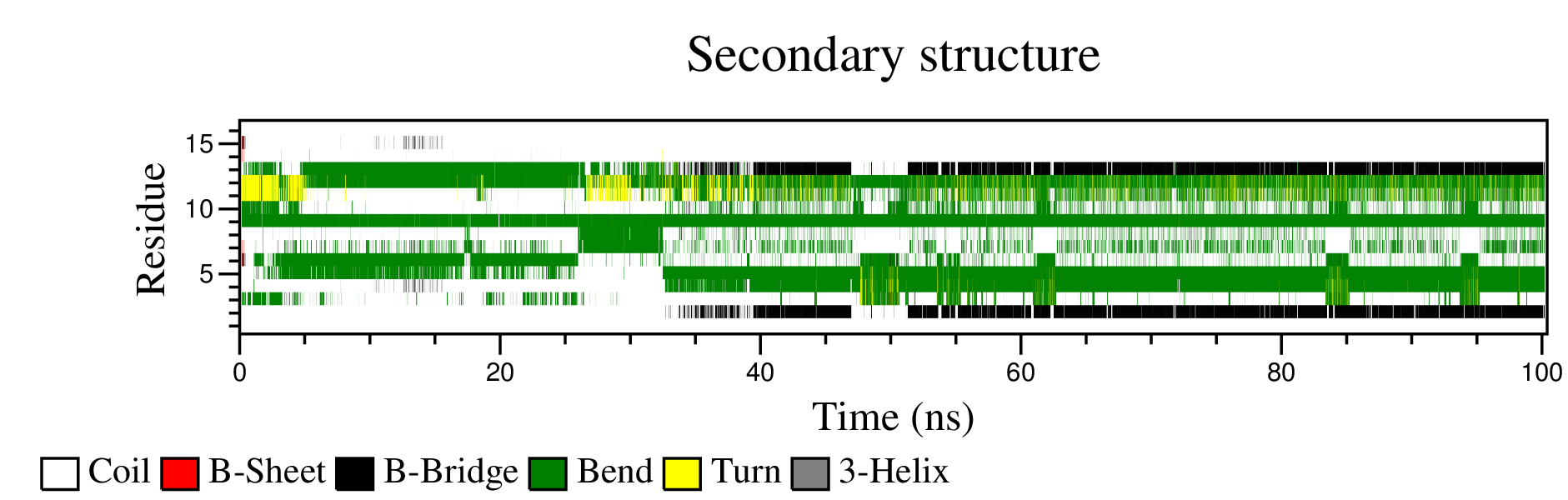


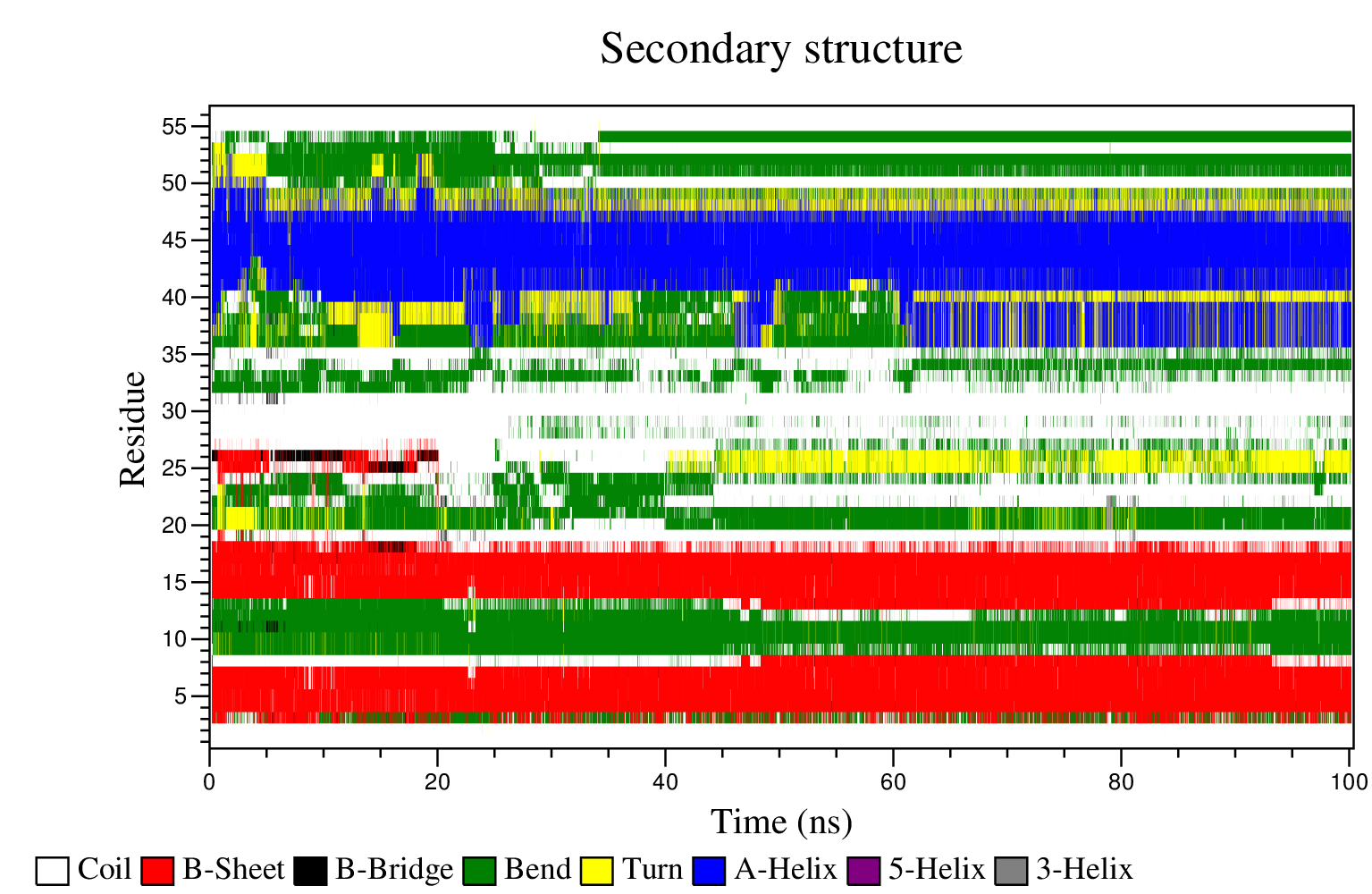


Figure S5: Secondary structural evolution of the random sequence variants of (a) the β-hairpin from Fig. S4b1 (top); and (b) Protein G from Fig. S4c1 (bottom) over 100 ns of MD simulation, as calculated by DSSP. Their secondary structures are mostly composed of coils and bends, and share little resemblance with the corresponding structure of native sequences.

**Movie S1**: One representative trajectory of the folding of the reverse sequence of protein Trp-cage.
